# Metabolic flux analysis in hiPSC-CMs reveals insights into cardiac dysfunction in propionic acidemia Eva Richard

**DOI:** 10.21203/rs.3.rs-5874705/v1

**Published:** 2025-01-28

**Authors:** Eva Richard, Hannah Marchuk, Mar Álvarez, Wentao He, Xiaoxin Chen, Lourdes R. Desviat, Guo-Fang Zhang

**Affiliations:** Universidad Autónoma de Madrid: Universidad Autonoma de Madrid; Duke University; Universidad Autónoma de Madrid: Universidad Autonoma de Madrid; Duke University; Coriell Institute for Medical Research; Universidad Autónoma de Madrid: Universidad Autonoma de Madrid; Duke University

**Keywords:** propionic acidemia, human induced pluripotent stem cell-derived cardiomyocytes, metabolic flux, glucose metabolism, fatty acid metabolism, cardiac diseases

## Abstract

Propionic acidemia is an inborn error of metabolism caused by mutations in either the *PCCA* or *PCCB* genes. Patients with propionic acidemia experience a range of complications, including life-threatening cardiac dysfunctions. However, the pathological mechanisms underlying propionic acidemia-associated cardiac diseases remain largely unknown. To gain insights into the metabolic alterations in propionic acidemia, we studied human induced pluripotent stem cell-derived cardiomyocytes generated from a patient with propionic acidemia with two pathogenic PCCA mutations (*p.Cys616_Val633del* and p.Gly477Glufs9*) and from a healthy individual. Using stable isotope-based metabolic flux analysis, we confirmed that the *PCCA* mutations lead to impaired propionyl-CoA carboxylase activity in human induced pluripotent stem cell-derived cardiomyocytes. In addition to being converted to propionylcarnitine, the accumulated propionyl-CoA can also be hydrolyzed to propionate and exported out of the cell, serving as a secondary “pressure valve” to regulate cellular propionyl-CoA levels. Interestingly, the deficiency of propionyl-CoA carboxylase was found to shift fuel metabolism from fatty acid oxidation to increased glucose metabolism human in induced pluripotent stem cell-derived cardiomyocytes from patients with propionic acidemia. This metabolic switch is less energy-efficient and may contribute to the development of chronic cardiac dysfunction in patients with propionic acidemia.

## Introduction

Propionic acidemia (PA) is an inborn error of metabolism inherited in an autosomal recessive manner [18, 36, 37, 59]. Mutations in either the *PCCA* or *PCCB* gene result in the malfunction of propionyl-CoA carboxylase (PCC), a critical mitochondrial enzyme. The global incidence of PA varies, ranging from 1 in 50,000 to 1 in 100,000 live births. The adoption of tandem mass spectrometry for neonatal screening has significantly increased the detection rate of PA cases [49].

PCC is a crucial mitochondrial enzyme, though it remains relatively understudied [56]. Whole-body PCC knockout in mouse models has been shown to be neonatal lethal [10, 21, 25, 40]. Patients with PA, often present symptoms in early infancy such as poor feeding, vomiting, and low muscle tone [2, 32]. As the disease progresses, patients may develop a variety of complications, including life-threatening cardiac dysfunctions [30, 31, 55]. Understanding the pathological mechanisms underlying PA-associated cardiac diseases is an urgent and critical need.

PCC catalyzes the carboxylation of propionyl-CoA to methylmalonyl-CoA, which is subsequently converted to succinyl-CoA and enters the tricarboxylic acid (TCA) cycle as part of an anaplerotic process [8, 27, 28, 39, 41]. Malfunctioning PCC disrupts propionyl-CoA metabolism, leading to the accumulation of propionyl-CoA and its metabolites, including methylcitrate, 3-hydroxypropionate, and maleic acid [51, 54]. These accumulated metabolites have been reported to inhibit the TCA cycle and mitochondrial energy production in *in vitro* enzyme assays using high concentrations of these metabolites [6, 12, 45, 46, 54]. Additionally, oxidative stress, disruption of potassium channels, and altered miRNA expression have been implicated in the cardiac dysfunction observed in patients with PA [7, 11, 17, 19, 20, 44, 47, 49].

Propionyl-CoA, an intracellular metabolite, cannot cross the plasma membrane [52]. Instead, accumulated propionyl-CoA is converted to propionylcarnitine, which can be released into the blood and urine [9, 14]. L-carnitine has been shown to effectively treat PA and is recommended as a supplement for patients [15, 42, 43]. The conversion of propionyl-CoA to propionylcarnitine is considered a critical metabolic pathway for the disposal of accumulated propionyl-CoA, with elevated propionylcarnitine serving as a biomarker for PA. However, PA is also associated with elevated levels of circulating propionate, which is partly due to reduced hepatic disposal [53]. The exact source of circulating propionate remains unclear. While it is known to originate from the microbiome, it is uncertain whether it might also arise from intracellular propionyl-CoA within the host. This could represent a secondary regulatory mechanism for managing intracellular propionyl-CoA, in addition to its conversion to propionylcarnitine.

Previously, we reported that supraphysiological levels of propionate dramatically increase propionyl-CoA and deplete free Coenzyme A (CoA) in perfused rat hearts [51]. This CoA depletion causes a metabolic shift from the high energy-efficient fatty acid oxidation to low energy-efficient glucose metabolism [51]. However, in the *Pcca*^−/−^(A138T) mouse model of PA, CoA or carnitine depletion in the heart occurs only upon the acute administration of high doses of propionate (500 mg/kg) [24]. This highlights the need for a more representative PA model to investigate the pathological mechanisms underlying cardiac diseases associated with PA.

Animal experiments often face limitations in their applicability to humans. In this study, we utilized human induced pluripotent stem cell-derived cardiomyocytes (hiPSC-CMs) from a control individual and a PA patient to investigate metabolic alterations and better understand the pathological mechanisms of PA. Using stable isotope-based metabolic flux analysis, we confirmed the metabolic phenotype of PCC deficiency in PA patient-derived hiPSC-CMs. Our experiments demonstrated that accumulated propionyl-CoA can be hydrolyzed to propionate, which is then exported from the cell as an additional “pressure valve.” Furthermore, PA hiPSC-CMs exhibited a metabolic switch from energy-efficient fatty acid oxidation to energy-inefficient glucose metabolism. This shift in fuel utilization may be a key pathological factor contributing to the cardiac dysfunction observed in PA patients.

## Methods

### Maintenance of hiPSC lines

The hiPSC lines utilized in this study included: (i) a PCCA-deficient hiPSC line (PCCA23-FiPS4F6 or UAMi001-A), created by reprogramming fibroblasts from a patient with *PCCA* gene mutations (c.1899 + 4_1899 + 7delAGTA; p.(Cys616_Val633del) and c.1430-?_1643+?del; p.(Gly477Glufs*9)) via Sendai virus; and (ii) a normal control hiPSC line (FIPS Ctrl2-SV4F-1) acquired from the National Bank of Cell Lines at the Carlos III Health Institute (ISCIII, Madrid, Spain).

These human iPSC lines were cultured on 60 mm dishes coated with Matrigel (hESC-quali ed matrix, Corning, New York, NY, USA) and maintained in mTESR^™^ Plus medium (StemCell^™^ Technologies, Vancouver, BC, Canada), with media changes every other day. The hiPSCs were passaged every four days using ReleSR^™^ (StemCell^™^ Technologies) and 10 μM Rock inhibitor (StemCell^™^ Technologies) at a splitting ratio of 1:3 to 1:5.

### Differentiation of hiPSCs into cardiomyocytes

hiPSCs cultured in mTESR^™^ Plus medium were dissociated into single cells using StemPro Accutase (Gibco, Waltham, MA, USA). A total of 1×10^6^ cells in 1.5 ml of mTESR^™^ Plus medium enriched with 10 μM Rock inhibitor were plated on Matrigel-coated 12-well plates. The differentiation into cardiomyocytes was conducted using the STEMdiff^™^ Cardiomyocyte Differentiation and Maintenance Kits (StemCell^™^ Technologies) following the guidelines provided by the manufacturer. The characterization of cardiomyocytes was achieved by analyzing the expression of various cardiac-specific markers, including cardiac troponin T, α-smooth muscle actin, GATA4, and α-actinin 2 through immunocytochemistry as previously described [5].

### Experimental conditions and metabolic treatments of hiPSC-derived cardiomyocytes

Control and PA hiPSC-derived cardiomyocytes were subjected to four experimental conditions to assess the impact of various metabolites: Experiment #1: hiPSC-CMs were cultured in RPMI/B27 medium incubated for four days at 37°C in a 5% CO_2_ atmosphere. Experiment #2 (Tracing experiment): hiPSC-CMs were cultured in RPMI/B27 medium with 1 mM [^13^C_3_]propionate (Sigma-Aldrich). Cells were incubated for two days at 37°C in a 5% CO_2_ atmosphere. Experiment #3 (Tracing experiment): hiPSC-CMs were cultured in RPMI/B27 medium without glucose and supplemented with 11 mM [^13^C_6_]glucose (Sigma-Aldrich) and incubated for two days at 37°C in a 5% CO_2_ atmosphere. Experiment #4 (Tracing experiment): hiPSC-CMs were cultured in RPMI/B27 with 0.4 mM [^13^C_16_]palmitate conjugated with BSA (Sigma-Aldrich) medium and incubated for two days at 37°C in a 5% CO_2_ atmosphere. After the incubation period, both the medium and the cell pellets were collected and frozen at −80°C for subsequent analysis.

### Short-chain fatty acids analysis by LC-MS/MS

An LC-MS/MS method was adapted to analyze short-chain fatty acids including propionate in media [53]. A 30-μl media sample was mixed with 30 μl internal standard (200 μM [2,2,2-^2^H_3_-1,2-^13^C_2_]aceate (D5 acetate), 20 μM [2,2,3,3,3-^2^H_5_]propionate (D5 propionate), 20 μM [2,2,3,3,4,4,4-^2^H_7_]butyrate (D7 butyrate), 20 μM [2,2,3,3,4,4,5,5,5-^2^H_9_]pentanoate (D9 pentanoate), and 20 μM [2,2,3,3,4,4,5,5,6,6,−^2^H11]hexanoate (D11 hexanoate)). Acetonitrile (1ml) was added to precipitate protein. The supernatant was transferred to a new Eppendorf vial and completely dried by nitrogen gas after samples were vortexed and centrifugated at 10000 ×g for 20 minutes. The dried residue was resuspended in 50 μl HPLC water and 20 μl 3-Nitrophenylhydrazine hydrochloride (EDC, 120 mM) and 20 μl (N-(3-Dimethylaminopropyl)-N′-ethylcarbodiimide (3-NPH, 200 mM) for derivatization at 40°C for 30 minutes. The reaction mixture was centrifuged for 10 minutes at 10,000 × g and the supernatant was transferred to an LC-MS/MS vial for analysis. LC-MS/MS was run with a Sciex QTRAP 6500^+^ MS connected with a Sciex AD UHPLC. An Agilent C18 column (Pursuit XRs C18 150 × 2.0 mm, 5 μm) was employed for separation at room temperature with a flow rate of 0.4 ml/min. A gradient method was conducted with two mobile phases. Mobile phase A was 98% H_2_O and 2% acetonitrile containing 0.1% formic acid. Mobile phase B was 98% acetonitrile and 2% H_2_O containing 0.1% formic acid. The gradient started with 2% B for the first 0.5 minutes and increased to 90% at 8 minutes. B was maintained at 90% for 4.5 minutes and returned to its initial condition within 0.5 minutes. Finally, the column was re-equilibrated for 9 minutes with the initial condition before the next injection. The injection volume was 3 μl. MRM in negative mode was used for short-chain fatty acids assay. The MS/MS parameters were set at the following: curtain gas: 35 psi, source temperature: 600°C, Gas 1: 55 psi, Gas 2: 55 psi, CAD: 10, Ion spray voltage: −4500 V, EP: −10 V, and CXP: −14.

### Cell pellets metabolic profiling by GC-MS

A previously established GC-MS method was adopted to measure the isotope labeling of organic acids and amino acids in the cultured pellets [22–24, 51, 53, 57]. Brie y, approximately 1 million cells were spiked with 2 nmol of norvaline and 0.2 nmol [^2^H_9_]L-carnitine or mixed stable isotope labeled metabolites as internal standards and then subjected to sonication extraction with 1 ml methanol for 3 minutes. The samples were centrifuged for 20 minutes. The upper phase, approximately 500 μl in volume, was transferred to a fresh Eppendorf vial and subsequently evaporated using nitrogen gas. The resulting dried residues underwent sequential derivatization with methoxylamine hydrochloride and N-tert-butyldimethylsilyl-N-methyltri uoroacetamide (TBDMS). Specifically, 40 μl of methoxylamine hydrochloride (2% (w/v) in pyridine) was added to the dried residues, followed by incubation for 90 minutes at 40°C. Subsequently, 20 μL of TBDMS with 1% tert-butylchlorodimethylsilane was added, and the mixture was incubated for an additional 30 minutes at 80°C. The derivatized samples were then centrifuged for 10 minutes at 12,000 × g, and the supernatants were transferred to GC vials for further analysis. For GC/MS analysis, we employed an Agilent 7890B GC system with an Agilent 5977A Mass Spectrometer, following the methodology described in our previous work. Specifically, 1 μl of the derivatized sample was injected into the GC column. The GC temperature gradient began at 80°C for 2 minutes, increased at a rate of 7°C per minute to 280°C, and was maintained at 280°C until the 40-minute run time was completed. The ionization was conducted via electron impact (EI) at 70 eV, with Helium flow at 1 mL/min. Temperatures of the source, the MS quadrupole, the interface, and the inlet were maintained at 230°C, 150°C, 280°C, and 250°C, respectively. Mass spectra (m/z) in the range of 50 to 700 were recorded in mass scan mode.

### Acylcarnitines profile by LC-MS/MS

A 100 μl medium sample or ~ 1 million cell pellets were used for acylcarnitine assay with the spiked internal standard (20 μl 0.01 mM D9 carnitine). The detailed LC-MS/MS method for the acylcarnitine pro le was described in our previous work [23, 24, 51, 53]. The pellet sample extracts (500 μl) from the previous sample preparation were completely dried using nitrogen gas. The medium samples were deproteinized by adding 500 ul methanol and 500 ul acetonitrile. After centrifugation at 12000 g for 15 minutes, the supernatants were completely dried using nitrogen gas. The dried residues were then methylated with a 3 M HCl methanol solution (100 μl) at 50°C for 25 minutes. After methylation, the samples were once again dried completely using nitrogen gas and then reconstituted in 20 μl of methanol and 60 μl of water. The derivatized samples were subsequently analyzed using an LC-QTRAP 6500^+^-MS/MS (Sciex, Concord, Ontario). A gradient HPLC method with two mobile phases (mobile phase A was 98% water with 2% acetonitrile and 0.1% formic acid and mobile phase B was 98% acetonitrile with 2% H_2_O and 0.1% formic acid) was adopted to run with an Agilent Pursuit XRs 5 C18 column (150 × 2.0 mm). The gradient started with 0% B within first 2 minutes and then increased to 80% at 13 minutes. The column was washed out by 90% B for 4 minutes and equilibrated with initial condition (2% B) for 5 minutes before next injection. The flow rate was 0.4 ml/minute and the column oven was set at room temperature. The injection volume was 2 μl. The parameters for Sciex QTRAP 6500 + mass spectrometry were optimized as following: DP: 33 V, EP 10 V, CXP: 10 V, source temperature: 680°C, gas 1: 65, gas 2: 65, curtain gas: 35, CAD: 10, and ion spray voltage: 5500 V. The Q1 of all the methylated acylcarnitines was scanned from m/z 218 to m/z 444 with the same fragment (Q3) at m/z 99. L-carnitine had the ion transition of Q1 (m/z 176) and Q3 (m/z 85 or m/z 117). [^2^H_9_]L-carnitine has the shifted Q1 at m/z 179 or m/z 185 with the same Q3 at m/z 85 or m/z 117.

### Medium glucose assay by LC-Q-Exactive-MS

Glucose in the medium was assayed according to our previous method. Brie y, a 10 μl of medium sample was added to a tube prior to folch extraction using the following solvents: 200 μl methanol, 200 μl distilled H_2_O, and 200 μl chloroform. The sample mixture was vortexed and centrifuged for 20 minutes at 10,000 × g at 4°C. The upper phase (~ 350 μl) was dried completely by nitrogen gas at 37°C. The dried residue was resuspended in 60 μl distilled water, vortexed, and placed in an autosampler vial for LC-MS analysis.

LC-Q-Exactive^+^-Orbitrap-MS was used for the final quantitation in this work. The Vanquish Binary Pump was used to deliver the mobile phase (98% H_2_O and 2% methanol containing 0.01% formic acid) at a flow rate of 0.3 ml/min in isocratic elution mode. The column was a Microsorb-MV C18 column (100 × 4.6 mm, 3 μm) with a C18 guard column and was kept at 40°C in the column oven compartment. The autosampler was maintained at 5°C, and the injection volume was 1 μl. The total running time is 10 minutes. The parameters for Q-Exactive^+^-MS equipped with a HESI probe: heat temperature: 425°C; sheath gas: 30, auxiliary gas, 13; sweep gas, 3; spray voltage, 3.5 kV for positive mode; the capillary temperature was set at 320°C, and S-lens was 45. A full scan range was set at 60 to 900 (m/z). The resolution was set at 70,000 (at m/z 200). The maximum injection time (max IT) was 200 ms. Automated gain control (AGC) was targeted at 3 × 10^6^ ions.

### Statistics

All cell experiments were conducted using two differentiations with a total of n = 4 biological replicates. Measured mass isotopologues distributions expressed as mol percent were corrected for natural enrichment [16, 50]. M0, M1, M2, ..., Mn denote the isotopologues of molecules containing n heavy atoms. Statistical differences were analyzed using Prism software. A Student’s t-test was used for comparisons between two groups, while a two-way ANOVA followed by a post hoc Tukey test was conducted for comparisons among more than two groups.

## Results

### Metabolic profile of human induced pluripotent stem cell-derived cardiomyocytes from a control Individual and a PCCA-deficient patient

**Figure 1:**
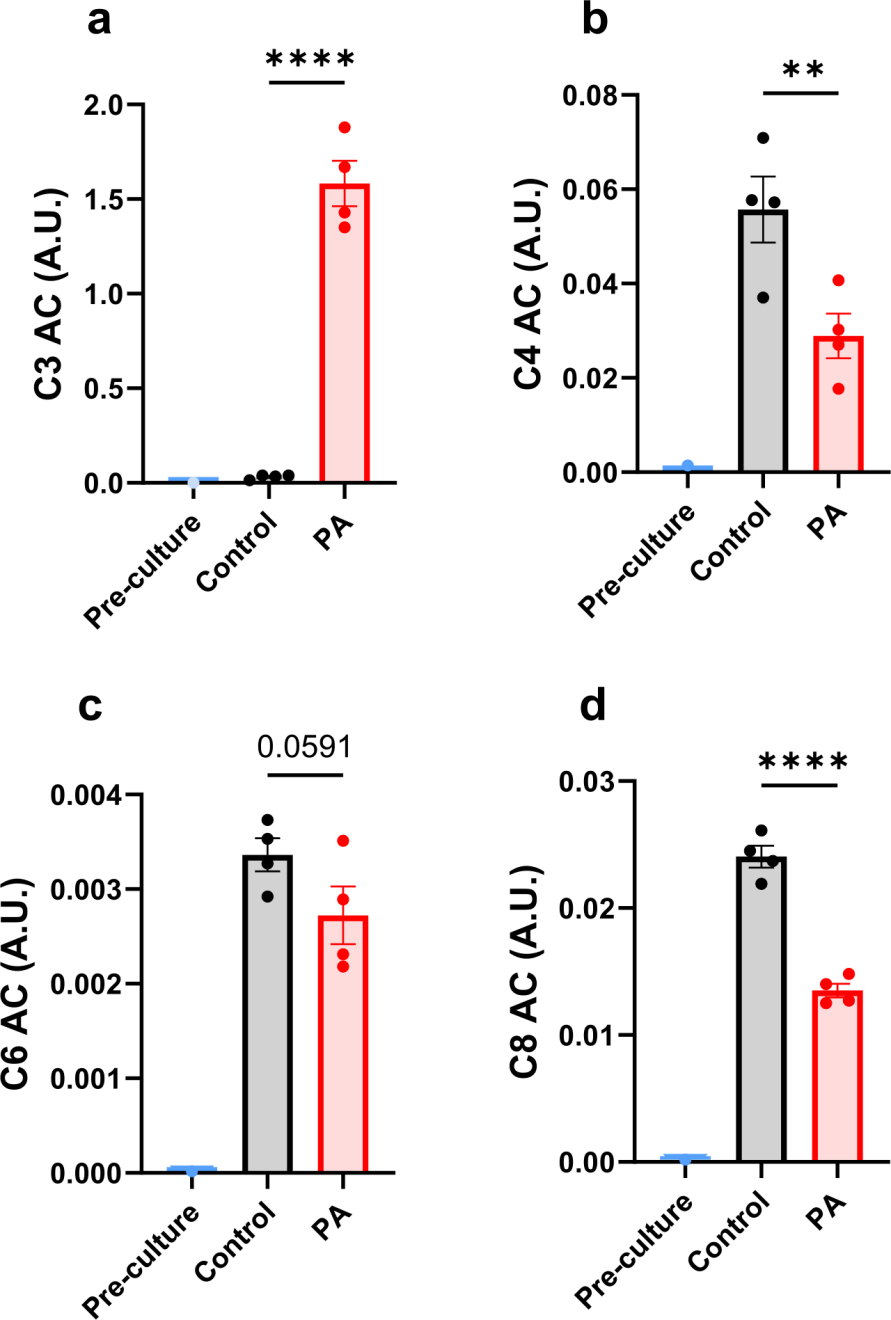
Release of acylcarnitines from hiPSC-CMs into the culture medium. (**a-d**) Levels of propionylcarnitine (C3 AC), butyrylcarnitine (C4 AC), hexanoylcarnitine (C6 AC), and octanoylcarnitine (C8 AC) in the culture medium after a 4-day incubation with RPMI/B27 medium. Data are presented as mean ± SE, with N=4 per group. ** and **** indicate p-values < 0.01 and < 0.001, respectively.

Metabolic pro ling was performed on control and PCCA-deficient hiPSC-CMs using both cell pellets and cultured media samples. PCC deficiency significantly increased propionylcarnitine (C3 AC) in the cultured media (Fig. 1A) and led to the accumulation of cellular methylcitrate (Supplemental Fig. 1A). Interestingly, medium-chain acylcarnitines were found to be lower in the cultured media from the PCCA-deficient group (Figs. 1B–1D). This decrease in acylcarnitines may indicate reduced fatty acid oxidation or a redistribution of acylcarnitines toward increased propionylcarnitine synthesis. In line with this, there was a trend toward a reduction in cellular L-carnitine (p = 0.076, Supplemental Fig. 1B). The accumulation of propionylcarnitine, along with the reduction of free carnitine, could alter fuel metabolism in the cardiomyocytes, which depend on proper fuel metabolism for mechanical contraction.

### PCC deficiency disrupts propionate and propionyl-CoA metabolism in hiPSC-CMs

**Figure 2:**
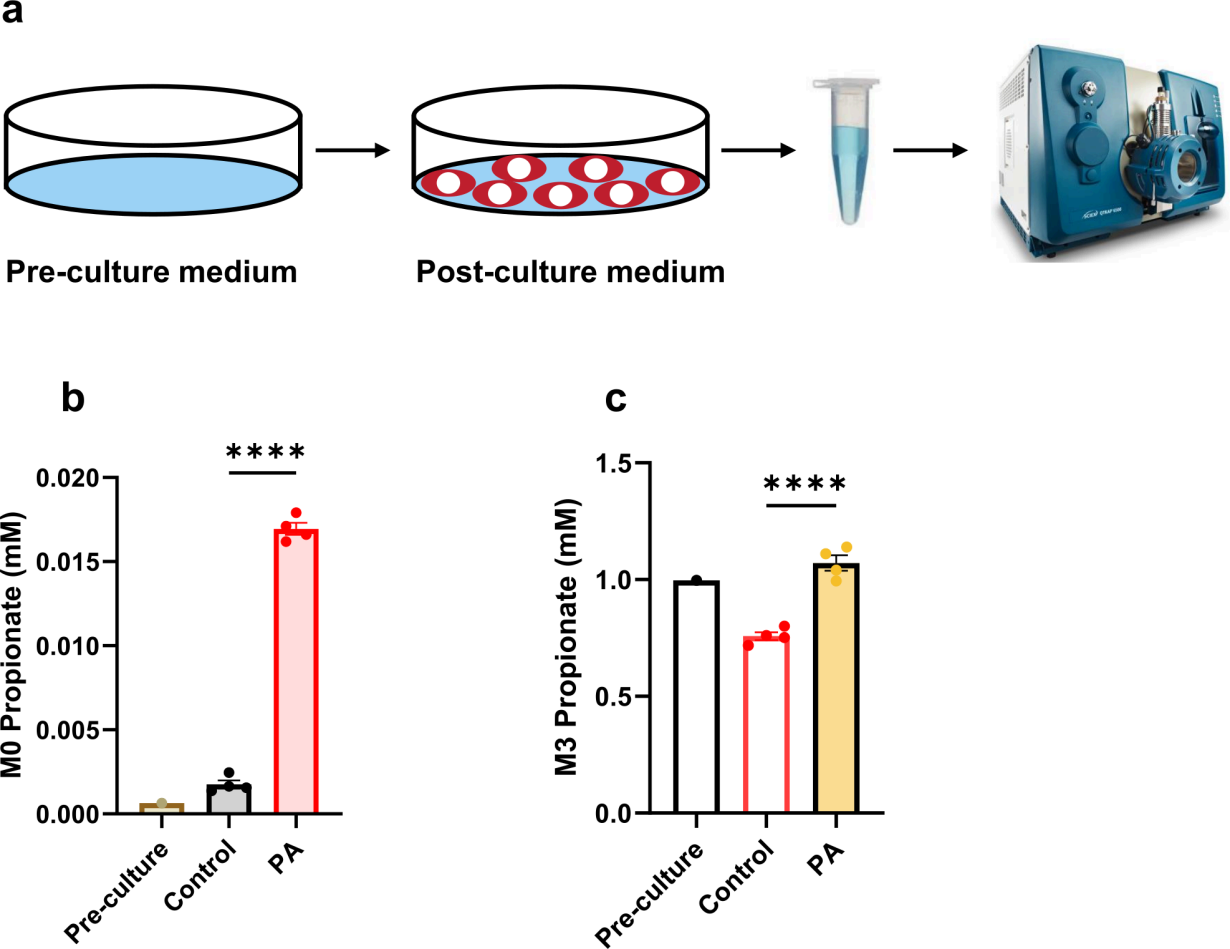
Effect of PCC deficiency on [^13^C_3_]propionate consumption**.** (**a**) Schematic representation of the experimental design and procedure. (**b**) Levels of unlabeled propionate (M0 propionate) in the pre-culture medium and post-culture medium of hiPSC-CMs derived from healthy controls (Control) and PA patients (PA). (**c**) Residual [^13^C_3_]propionate (M3 propionate) in the culture medium after 2 days of incubation with 1 mM [^13^C_3_]propionate in RPMI/B27 medium, comparing hiPSC-CMs from Control and PA groups. Data are presented as mean ± SE, with N=4 per group. **** indicates p-values < 0.001.

One of the reliable biomarkers of PA is elevated circulating propionate, which can reach millimolar levels in PA patients [26, 53]. However, this biomarker is not often reported due to challenges with analytical methodology. Our previous work suggests that impaired hepatic disposal of propionate leads to increased circulating levels, which may elevate the risk of cardiac disease [53]. In the present study, we aimed to investigate the impact of PCC deficiency on propionate metabolism in cardiomyocytes derived from both control and PCCA-deficient hiPSC. After culturing the cells for 48 hours with 1 mM [^13^C_3_]propionate, both pre-culture and post-culture media were collected for propionate quantification (Fig. 2A). PCCA-deficient hiPSC-CMs released 9.6 times more unlabeled propionate compared to control hiPSC-CMs (Fig. 2B). The released unlabeled propionate is likely generated endogenously, probably from hydrolysis of propionyl-CoA, which is elevated in PA patient-derived hiPSC-CMs. After 48 hours of culturing, [^13^C_3_]propionate was consumed by 25% in the control hiPSC-CMs (Fig. 2C). In contrast, [^13^C_3_]propionate levels in the medium remained almost unchanged in PA hiPSC-CMs (Fig. 2C). This strongly suggests that the PCC deficiency not only affects propionyl-CoA metabolism but also disrupts propionate metabolism, consistent with our previous findings in the livers of *Pcca*^−/−^(A138T) mice [53].

**Figure 3:**
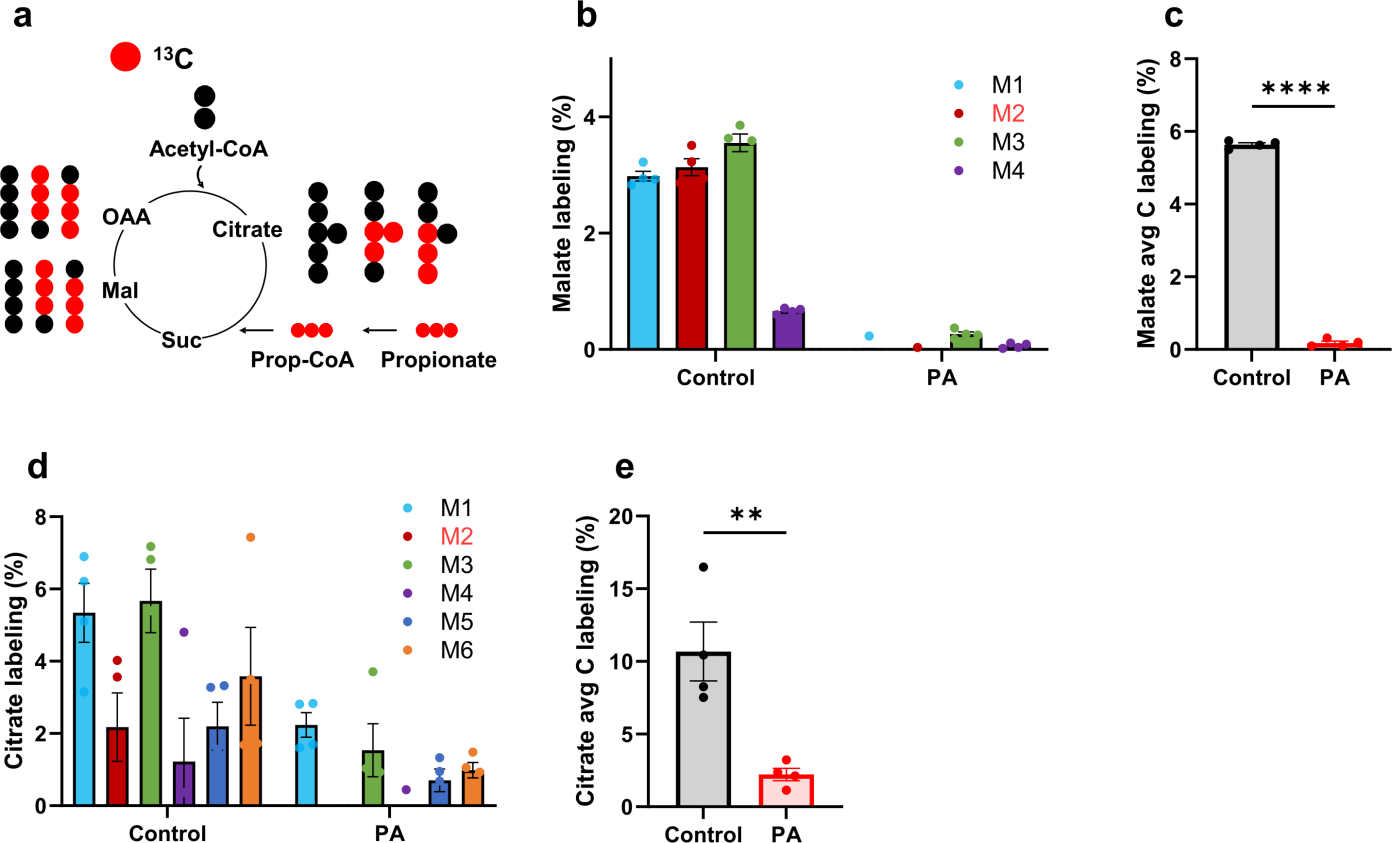
PCC deficiency reduces [^13^C_3_]propionate metabolism**.** (**a**) Simplified schematic of the [^13^C_3_]propionate tracing pathway during the first round of TCA cycle entry. (**b-c**) Stable isotopomer labeling and average carbon (C) labeling of malate. (**d-e**) Stable isotopomer labeling and average carbon (C) labeling of citrate. hiPSC-CMs from control individual and PA patient were incubated with 1 mM [^13^C_3_]propionate for 48 hours. Data are presented as mean ± SE, with N=4 per group. ** and **** represent p-values < 0.01 and < 0.001, respectively.

[^13^C_3_]Propionate, used as a tracer, allows for tracking both propionate and propionyl-CoA metabolism [24]. TCA cycle intermediates are downstream metabolites of propionyl-CoA, and stable isotope labeling of these intermediates reflects propionyl-CoA metabolism (Fig. 3A). The stable isotopomer labeling and average carbon labeling of malate and citrate are shown in Figs. 3B–3E. The low labeling of malate and citrate in PA hiPSC-CMs con rms that the PCCA mutation impairs PCC activity and demonstrates that this disrupts propionyl-CoA anaplerosis into the TCA cycle. The dramatic reduction in labeling of TCA cycle intermediates is further corroborated by other metabolite labeling data (Supplemental Fig. 2).

### PCC deficiency enhances glucose metabolism in hiPSC-CMs

**Figure 4:**
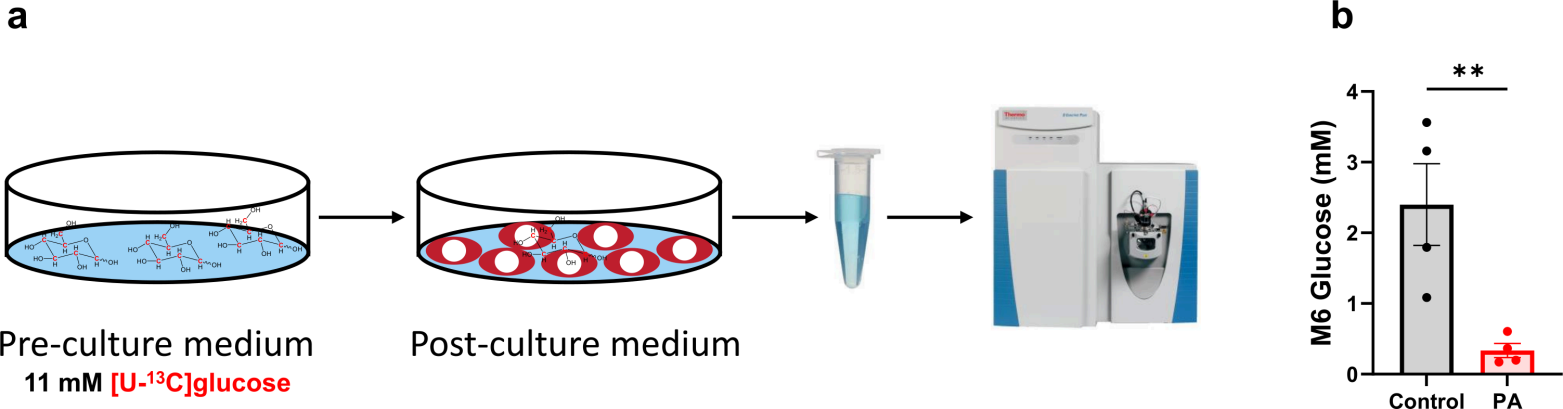
PCC deficiency increases [^13^C_6_]glucose consumption**.** (**a**) Schematic representation of the experimental design and procedure for measuring [^13^C_6_]glucose consumption from pre-culture and post-culture media. (**b**) [^13^C_6_]Glucose concentrations in the culture media after 48 hours of incubation with hiPSC-CMs derived from a healthy control individual and a PA patient, using 11 mM [^13^C_6_]glucose. Data are presented as mean ± SE, with N=4 per group. ** indicates p-values < 0.01.

Approximately 25% or more of patients with PA develop cardiac diseases, although the pathological mechanisms remain largely unknown [34]. Fuel metabolism is crucial for cardiac function, as it sustains energy production required for mechanical contraction. To better understand the metabolic alterations in PA, we investigated the major fuel metabolism pathways in PA hiPSC-CMs. We replaced unlabeled glucose (11 mM) with 11 mM [^13^C_6_]glucose in the medium to trace glucose metabolism. First, we measured the consumption of [^13^C_6_]glucose by quantifying the levels of [^13^C_6_]glucose in both pre- and post-culture media using LC-Q Exactive^+^-MS (Fig. 4A). After 48 hours of incubation, [^13^C_6_]glucose levels were much lower in the medium of PA hiPSC-CMs compared to control hiPSC-CMs (Fig. 4B), indicating that PA hiPSC-CMs metabolize [^13^C_6_]glucose at a higher rate than control cells.

**Figure 5:**
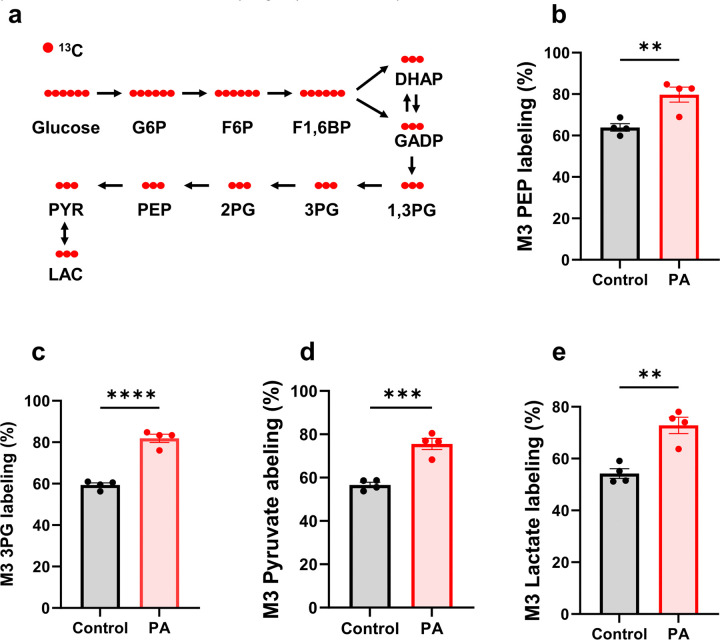
PCC deficiency enhances [^13^C_6_]glucose glycolysis**.** (**a**) Schematic representation of [^13^C_6_]glucose metabolism through glycolysis, showing the labeling pathway. G6P, F6P, DHAP, GADP, 1,3PG, 2PG, PYR, and LAC represent glucose-6-phosphate, fructose-6-phosphate, fructose-1,6-bisphosphate, dihydroxyacetone phosphate, glyceraldehyde 3-phosphate, 1,3-bisphosphoglycerate, 2-phosphoglycerate, pyruvate, and lactate, respectively. (**b-e**) M3 isotopomer labeling of glycolytic intermediates, including phosphoenolpyruvate (PEP), 3-phosphoglycerate (3PG), pyruvate, and lactate, in hiPSC-CMs from the control individual and the PA patient after 48 hours of culture with 11 mM [^13^C_6_]glucose. Data are presented as mean ± SE, with N=4 per group. **, ***, and **** indicate p-values < 0.01, < 0.005, and < 0.001, respectively.

Using ^13^C labeled tracer, we traced the downstream metabolites of glucose to assess glucose metabolic rate through stable isotopomer analysis (Fig. 5A). The stable isotope labeling of glycolytic intermediates—such as 3-phosphoglycerate (3PG), phosphoenolpyruvate (PEP), pyruvate, and lactate—was measured (Figs. 5B–5E). The stable isotope labeling of these intermediates was significantly higher in PA hiPSC-CMs, reaching 80%, compared to 60% in control hiPSC-CMs. This con rms that glucose metabolism is elevated in PA-derived cardiomyocytes.

**Figure 6:**
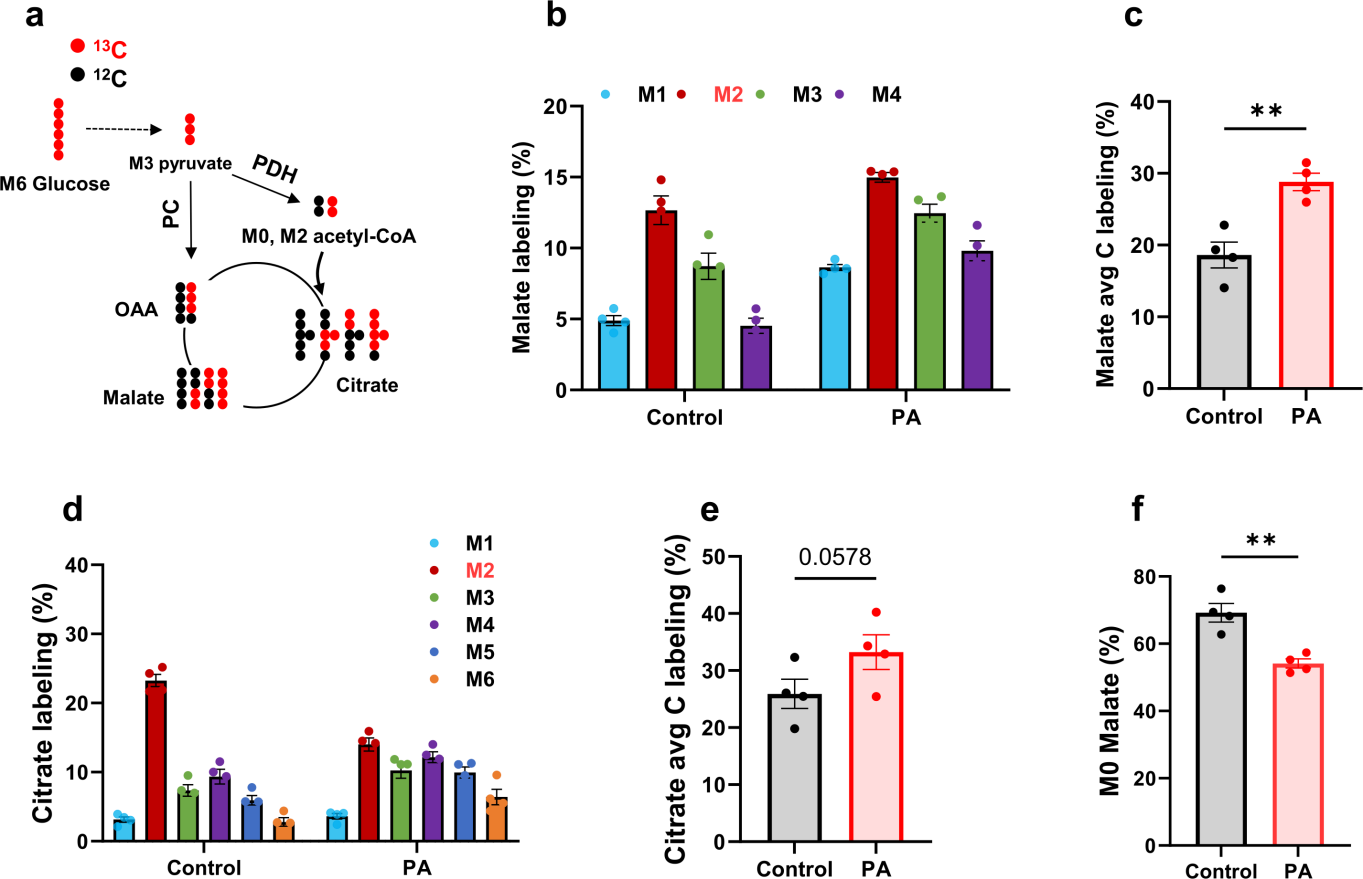
PCC deficiency enhances [^13^C_6_]glucose metabolism through the TCA cycle**.** (**a**) Schematic representation of [^13^C_6_]glucose metabolism through the first round of the TCA cycle, showing [^13^C_3_]pyruvate entry via pyruvate dehydrogenase (PDH) and pyruvate carboxylase (PC). Key intermediates include acetyl-CoA, citrate, malate and oxaloacetate (OAA). (**b-e**) Stable isotopomer labeling and average carbon (C) labeling of malate and citrate derived from [^13^C_6_]glucose in hiPSC-CMs from the control individual and the PA patient after 48 hours of culture. (**f**) Percentage of unlabeled malate in the same samples. Data are presented as mean ± SE, with N=4 per group. ** indicates p-values < 0.01.

Further downstream in the glucose metabolic pathway, TCA cycle intermediates were labeled by [^13^C_6_]glucose (Fig. 6A). Figures 6B–6E show the stable isotopomer labeling and average carbon labeling of malate and citrate in control and PA hiPSC-CMs. Consistent with the increased glucose metabolism in PA hiPSC-CMs, the stable isotope labeling of malate was significantly higher in PA than in control cells. Additionally, the shift to a higher isotopomer of citrate in PA hiPSC-CMs demonstrates increased metabolic flux of glucose into the TCA cycle (Fig. 6D). The lower M2 citrate in PA hiPSC-CMs also supports the finding of reduced unlabeled oxaloacetate/malate (Fig. 6F), as M2 citrate is primarily derived from M0 oxaloacetate and M2 acetyl-CoA (M2 citrate = M0 oxaloacetate × M2 oxaloacetate). This pattern was also observed in other detected TCA cycle intermediates (Supplemental Fig. 3).

Interestingly, M3 malate was the second most abundant isotopomer after M2 (Fig. 6B). The minor amount of M3 malate could be generated from multiple turns of the TCA cycle after M2 acetyl-CoA enters the cycle (Supplement Fig. 4). M2 malate and M3 malate are primarily derived from M2 acetyl-CoA via pyruvate dehydrogenase and M3 pyruvate via pyruvate carboxylase, respectively. The increased glucose metabolism in PA hiPSC-CMs is likely driven by enhanced metabolic flux through both pyruvate dehydrogenase and pyruvate carboxylase.

### Increased secretion of labeled acetate and unlabeled propionate in PA hiPSC-CMs

**Figure 7:**
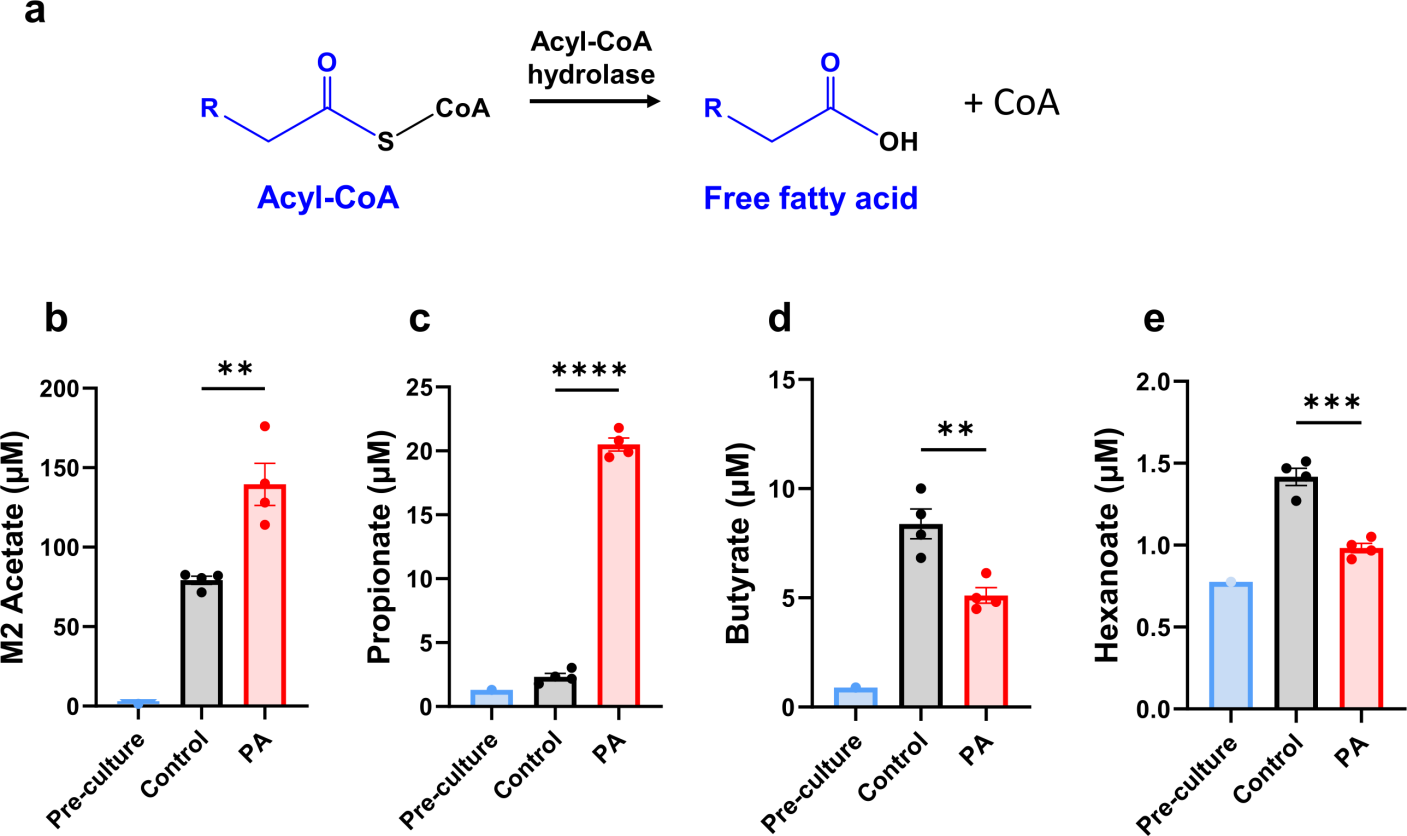
Short-chain fatty acids are hydrolyzed from acyl-CoAs in hiPSC-CMs**.** (**a**) Schematic representation of acyl-CoA hydrolase-mediated hydrolysis of acyl-CoAs into free fatty acids. (**b**) Concentration of M2 acetate in the cultured media from control and PA hiPSC-CMs after 48 hours of incubation with [^13^C_6_]glucose. (**c-e**) Concentrations of unlabeled propionate, butyrate, and hexanoate in the pre- and post-cultured media from control and PA hiPSC-CMs after 48 hours of incubation with [^13^C_6_]glucose. Data are presented as mean ± SE, with N=4 per group. ** and *** denote p-values < 0.01 and < 0.005, respectively.

Acyl-CoAs are intracellular metabolites that cannot cross the plasma membrane [52]. To prevent the accumulation of acyl-CoAs, which can be toxic due to their detergent-like effect, they are converted into their counterparts, acylcarnitines. This metabolic conversion serves as a protective mechanism. In the context of PA, carnitine supplementation can facilitate the conversion of accumulated propionyl-CoA to propionylcarnitine, helping to remove excess propionyl-CoA from cells. However, it remains unclear whether acyl-CoAs could also be hydrolyzed into free fatty acids, offering an additional means for the intracellular metabolites to escape cells and enter the bloodstream (Fig. 7A). In this study, we measured short-chain fatty acids in the cultured media and detected M2 acetate, derived from labeled M2 acetyl-CoA, in the media from [^13^C_6_]glucose (Fig. 7B). This confirmed the activity of acyl-CoA hydrolase, which plays a secondary role in regulating cellular acyl-CoA levels. The increased secretion of labeled acetate (M2 acetate) from [^13^C_6_]glucose in PA hiPSC-CMs also suggests enhanced glucose metabolism in these cells. As anticipated, accumulated propionyl-CoA led to the increased release of propionate into the medium (Fig. 7C) via acyl-CoA hydrolase, consistent with results from the [^¹³^C_₃_]propionate experiment (Fig. 2B). In contrast, the release of butyrate and hexanoate decreased, consistent with the reduction of medium-chain acylcarnitines (Figs. 7D, 7E, 1B–1E). This decrease in butyrate, hexanoate, and their corresponding acylcarnitines further supports the shift from fatty acid metabolism to glucose metabolism in PA hiPSC-CMs.

### Reduced fatty acid oxidation in PA hiPSC-CMs

**Figure 8:**
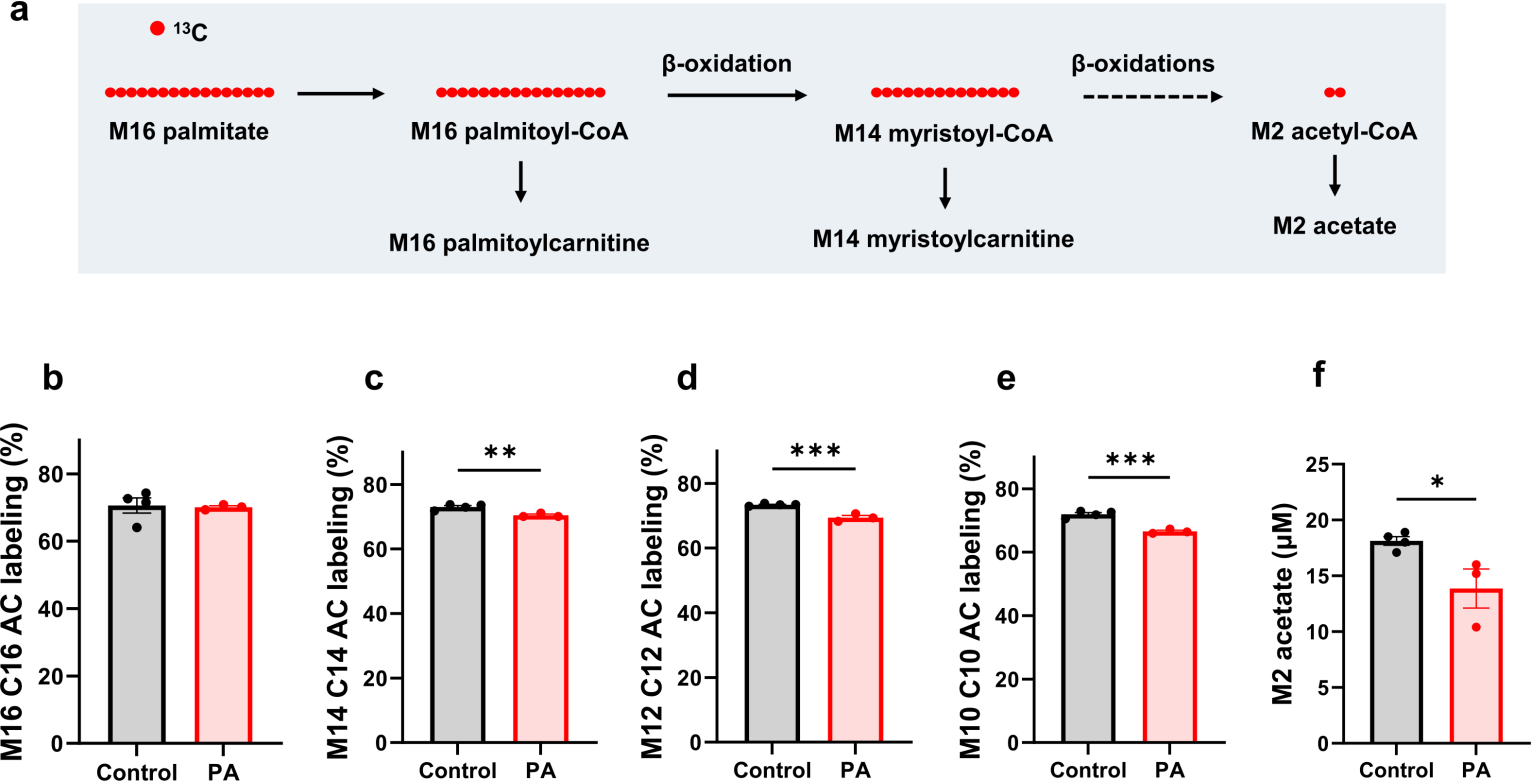
PCC deficiency reduces [^13^C_16_]palmitate metabolism**.** (**a**) Schematic representation of [^13^C_16_]palmitate metabolism. (**b-f**) Stable isotopomer labeling of M16 palmitoylcarnitine (M16 C16 AC), M14 myristoylcarnitine (M14 C14 AC), M12 lauroylcarnitine (M12 C12 AC), M10 caproylcarnitine (M10 C10 AC), and M2 acetate in the cultured media from control and PA hiPSC-CMs after 48 hours of incubation with 0.4 mM [^13^C_16_]palmitate. Data are presented as mean ± SE, with N=4 per group. *, **, and *** indicate p-values < 0.05, < 0.01, and < 0.005, respectively.

The increased lipid droplets have been observed in the heart from patients with PA [33]. A recent study using stable isotope techniques also demonstrated altered lipid metabolism in PA patients [48]. Given the increased glucose metabolism, we sought to investigate fatty acid metabolism in PA hiPSC-CMs using [^13^C_16_]palmitate. After 48 hours of culturing, we measured the labeling of metabolites derived from [^13^C_16_]palmitate (Fig. 8A). In the cultured medium samples, acylcarnitine intermediates from M16 palmitate were significantly lower in PA hiPSC-CMs (Figs. 8B–8E and Supplemental Figs. 5A-5C). Notably, M2 acetate labeling was also significantly lower in the cultured media of PA hiPSC-CMs (Fig. 8F). As observed in the [^13^C_6_]glucose tracing experiment, M2 acetate is derived from the hydrolysis of M2 acetyl-CoA, which is produced during the metabolism of both [^13^C_6_]glucose and [^13^C_16_]palmitate. However, in contrast to the [^13^C_6_]glucose experiment (Fig. 7B), M2 acetate labeling in PA hiPSC-CMs was significantly lower than in control cells when [^13^C_16_]palmitate was used as a tracer (Fig. 8F). Together, these findings suggest that PCC deficiency induces a fuel switch from fatty acid metabolism to increased glucose utilization.

Free carnitine plays a crucial role in transporting long-chain fatty acids into the mitochondria for complete β-oxidation. We observed a reduction, or a trend toward reduced, cellular L-carnitine in all experiments (Supplemental. Figure 1B and Supplemental Fig. 6). The L-carnitine deficiency in PA hiPSC-CMs impedes fatty acid oxidation, which in turn promotes increased glucose metabolism.

## Discussion

PA is a rare metabolic disorder caused by mutations in *PCCA* or *PCCB* genes, resulting in impaired propionyl-CoA metabolism. PA is typically diagnosed early in life, often within days of birth. If left unmanaged, PA can lead to severe complications, including cardiac dysfunction. The underlying mechanisms driving these cardiac complications are not yet fully understood. Chronic metabolic alterations associated with PA are believed to contribute to these complications.

Energy metabolism is crucial for the heart to perform mechanical work. Fatty acids are the heart’s preferred and most efficient fuel for ATP production. In failing hearts, however, fuel metabolism switches from fatty acids to increased glucose and ketone metabolism [13]. This shift may be particularly pronounced in PA patients due to several factors: (1) the depletion of L-carnitine and free CoA, caused by the accumulation of propionylcarnitine and propionyl-CoA, which impairs fatty acid oxidation, (2) the accumulation of lipid droplets in the heart, indicating disrupted fatty acid metabolism [4, 5], and (3) the inhibition of fatty acid oxidation during exercise in PA patients [48]. In this study, we observed that PA hiPSC-CMs exhibit a significant increase in glucose utilization and a decrease in fatty acid oxidation using stable isotope-based metabolic flux analysis. This chronic metabolic switch could impair cardiac energy metabolism and lead to progressive cardiac dysfunction, as the heart preferentially utilizes fatty acids for energy. Interestingly, while resting fatty acid oxidation in PA patients may not differ significantly from healthy individuals, PA patients are unable to fully utilize fatty acids during exercise when the whole-body switches to fatty acid oxidation [48].

The accumulation of propionylcarnitine in PA leads to decreased intracellular L-carnitine levels, particularly in tissues like the heart. One PA patient with fatal cardiomyopathy had low carnitine levels in the heart despite supplementation [38]. Reduced intracellular L-carnitine (Supplemental Fig. 1B and Supplemental Fig. 6) limits the transport of long-chain fatty acids into mitochondria for complete β-oxidation. Chronic reductions in fatty acid oxidation could trigger cardiac disease, as seen in patients with long-chain fatty acid β-oxidation disorders (LCFAOD), who frequently develop cardiomyopathy [29].

Propionate in plasma and urine is another reliable biomarker for PA. Short-chain fatty acids (SCFAs), typically produced by the microbiome, are usually at low levels in the portal vein of germ-free mice [23, 53]. The liver plays a key role in metabolizing and disposing of propionate, maintaining low levels ( ~ < 1 μM) [53]. However, it remains unclear whether SCFAs could be produced endogenously, given two key observations: (1) acetate, the most abundant SCFA in the blood, correlates well with acetyl-CoA levels, which are among the highest of all acyl-CoAs, and (2) propionate arises in PA due to propionyl-CoA accumulation. This raises the question of whether acyl-CoA hydrolysis might be another source of SCFAs.

Acyl-CoA hydrolysis, a process that releases free fatty acids that can be transported out of cells, is a potential regulatory mechanism that controls intracellular acyl-CoA levels and prevents their accumulation. Although the role of acyl-CoA hydrolases is understudied, they could play an essential role in regulating CoA for other metabolic events in the cell [1, 3]. In peroxisomes, acyl-CoAs are hydrolyzed into free fatty acids, which are then transported to mitochondria for complete β-oxidation [58]. We confirmed the acyl-CoA hydrolysis activity using stable isotope tracing in this study. Labeled acetate and unlabeled propionate, hydrolyzed from labeled acetyl-CoA and unlabeled propionyl-CoA, occur in mitochondria, where pyruvate oxidation to acetyl-CoA and propionyl-CoA metabolism predominantly take place. Free acetate from pyruvate metabolism could also be directly catalyzed by pyruvate dehydrogenase or 2-ketoglutarate dehydrogenase according to Liu et al.’s work [35]. However, the release of propionate, butyrate and hexanoate con rms the acyl-CoA hydrolase activity.

The physiological implications of propionyl-CoA hydrolysis into propionate in PA warrant further investigation. This hydrolysis could represent a second regulatory mechanism to control intracellular propionyl-CoA levels, serving as a “pressure valve” to mitigate the toxicity of accumulated propionyl-CoA in mitochondria.

This study is the first to utilize cardiomyocytes derived from patients with PA for metabolic discovery, emphasizing its strong clinical relevance. However, the authors acknowledge the following limitations: This is an *in vitro* experiment, which does not fully capture the complexity of metabolic regulation present *in vivo*. The *in vitro* conditions may not accurately reflect the physiological environment *in vivo*. These limitations highlight the need for future *in vivo* studies, which face the following challenges: (1) Metabolic data from the heart *in vivo* is often confounded by secondary metabolites originating from other organs. (2) Experimental findings using heart tissue from animal models may not directly translate to human patients.

In summary, using stable isotope tracing and hiPSC-CMs derived from PA patients, we provide the direct evidence that PA induces a fuel switch from fatty acids to glucose in cardiomyocytes derived from patients with PA. This shift may underlie the cardiac complications observed in PA. Additionally, we identify a second metabolic regulatory mechanism—acyl-CoA hydrolysis—that helps control propionyl-CoA levels in PA. This pathway may offer a potential therapeutic target for treating PA.

## Data Availability

Data will be made available from authors on request.

## References

[1] Agius L, Wright PD, Alberti KG (1987) Carnitine acyltransferases and acyl-CoA hydrolases in human and rat liver. Clin Sci (Lond) 73:3–10 doi:10.1042/cs0730003 2886246

[2] Al-Hamed MH, Imtiaz F, Al-Hassnan Z, Al-Owain M, Al-Zaidan H, Alamoudi MS, Faqeih E, Alfadhel M, Al-Asmari A, Saleh MM, Almutairi F, Moghrabi N, AlSayed M (2019) Spectrum of mutations underlying Propionic acidemia and further insight into a genotype-phenotype correlation for the common mutation in Saudi Arabia. Mol Genet Metab Rep 18:22–29 doi:10.1016/j.ymgmr.2018.12.004 30705822 PMC6349011

[3] Alexson SE, Nedergaard J (1988) A novel type of short- and medium-chain acyl-CoA hydrolases in brown adipose tissue mitochondria. J Biol Chem 263:13564–13571 2901416

[4] Alonso-Barroso E, Perez B, Desviat LR, Richard E (2021) Cardiomyocytes Derived from Induced Pluripotent Stem Cells as a Disease Model for Propionic Acidemia. Int J Mol Sci 22 doi:10.3390/ijms22031161 PMC7865492 33503868

[5] Alvarez M, Ruiz-Sala P, Perez B, Desviat LR, Richard E (2023) Dysregulated Cell Homeostasis and miRNAs in Human iPSC-Derived Cardiomyocytes from a Propionic Acidemia Patient with Cardiomyopathy. Int J Mol Sci 24 doi:10.3390/ijms24032182 PMC9916417 36768524

[6] Amaral AU, Cecatto C, Castilho RF, Wajner M (2016) 2-Methylcitric acid impairs glutamate metabolism and induces permeability transition in brain mitochondria. J Neurochem 137:62–75 doi:10.1111/jnc.13544 26800654

[7] Bodi I, Grunert SC, Becker N, Stoelzle-Feix S, Spiekerkoetter U, Zehender M, Bugger H, Bode C, Odening KE (2016) Mechanisms of acquired long QT syndrome in patients with propionic academia. Heart Rhythm 13:1335–1345 doi:10.1016/j.hrthm.2016.02.003 26854997

[8] Brunengraber H, Roe CR (2006) Anaplerotic molecules: current and future. J Inherit Metab Dis 29:327–331 doi:10.1007/s10545-006-0320-1 16763895

[9] Chalmers RA, Roe CR, Stacey TE, Hoppel CL (1984) Urinary excretion of l-carnitine and acylcarnitines by patients with disorders of organic acid metabolism: evidence for secondary insufficiency of l-carnitine. Pediatr Res 18:1325–1328 doi:10.1203/00006450-198412000-00021 6441143

[10] Chandler RJ, Chandrasekaran S, Carrillo-Carrasco N, Senac JS, Hofherr SE, Barry MA, Venditti CP (2011) Adeno-associated virus serotype 8 gene transfer rescues a neonatal lethal murine model of propionic acidemia. Hum Gene Ther 22:477–481 doi:10.1089/hum.2010.164 20950151 PMC3073074

[11] Chapman KA, Ostrovsky J, Rao M, Dingley SD, Polyak E, Yudkoff M, Xiao R, Bennett MJ, Falk MJ (2018) Propionyl-CoA carboxylase pcca-1 and pccb-1 gene deletions in Caenorhabditis elegans globally impair mitochondrial energy metabolism. J Inherit Metab Dis 41:157–168 doi:10.1007/s10545-017-0111-x 29159707 PMC5832583

[12] Cheema-Dhadli S, Leznoff CC, Halperin ML (1975) Effect of 2-methylcitrate on citrate metabolism: implications for the management of patients with propionic acidemia and methylmalonic aciduria. Pediatr Res 9:905–908 doi:10.1203/00006450-197512000-00008 127973

[13] Davila-Roman VG, Vedala G, Herrero P, de las Fuentes L, Rogers JG, Kelly DP, Gropler RJ (2002) Altered myocardial fatty acid and glucose metabolism in idiopathic dilated cardiomyopathy. J Am Coll Cardiol 40:271–277 doi:10.1016/s0735-1097(02)01967-8 12106931

[14] Di Donato S, Rimoldi M, Garavaglia B, Uziel G (1984) Propionylcarnitine excretion in propionic and methylmalonic acidurias: a cause of carnitine deficiency. Clin Chim Acta 139:13–21 doi:10.1016/0009-8981(84)90187-6 6723070

[15] Duran M, Ketting D, Beckeringh TE, Leupold D, Wadman SK (1986) Direct identification of propionylcarnitine in propionic acidaemia: biochemical and clinical results of oral carnitine supplementation. J Inherit Metab Dis 9:202–207 doi:10.1007/BF01799460 3091925

[16] Fernandez CA, Des Rosiers C, Previs SF, David F, Brunengraber H (1996) Correction of 13C mass isotopomer distributions for natural stable isotope abundance. J Mass Spectrom 31:255–262 doi:10.1002/(SICI)1096-9888(199603)31:3<255::AID-JMS290>3.0.CO;2-3 8799277

[17] Fulgencio-Covian A, Alonso-Barroso E, Guenzel AJ, Rivera-Barahona A, Ugarte M, Perez B, Barry MA, Perez-Cerda C, Richard E, Desviat LR (2020) Pathogenic implications of dysregulated miRNAs in propionic acidemia related cardiomyopathy. Transl Res 218:43–56 doi:10.1016/j.trsl.2019.12.004 31951825

[18] Galarreta Aima CI, Shchelochkov OA, Jerves Serrano T, Venditti CP (1993) Propionic Acidemia. In: Adam MP, Feldman J, Mirzaa GM, Pagon RA, Wallace SE, Amemiya A (eds) GeneReviews((R)). Seattle (WA) 22593918

[19] Gallego-Villar L, Perez-Cerda C, Perez B, Abia D, Ugarte M, Richard E, Desviat LR (2013) Functional characterization of novel genotypes and cellular oxidative stress studies in propionic acidemia. J Inherit Metab Dis 36:731–740 doi:10.1007/s10545-012-9545-3 23053474

[20] Gallego-Villar L, Rivera-Barahona A, Cuevas-Martin C, Guenzel A, Perez B, Barry MA, Murphy MP, Logan A, Gonzalez-Quintana A, Martin MA, Medina S, Gil-Izquierdo A, Cuezva JM, Richard E, Desviat LR (2016) In vivo evidence of mitochondrial dysfunction and altered redox homeostasis in a genetic mouse model of propionic acidemia: Implications for the pathophysiology of this disorder. Free Radic Biol Med 96:1–12 doi:10.1016/j.freeradbiomed.2016.04.007 27083476

[21] Guenzel AJ, Hofherr SE, Hillestad M, Barry M, Weaver E, Venezia S, Kraus JP, Matern D, Barry MA (2013) Generation of a hypomorphic model of propionic acidemia amenable to gene therapy testing. Mol Ther 21:1316–1323 doi:10.1038/mt.2013.68 23648696 PMC3708067

[22] He W, Berthiaume JM, Previs S, Kasumov T, Zhang GF (2023) Ischemia promotes acyl-CoAs dephosphorylation and propionyl-CoA accumulation. Metabolomics 19:12 doi:10.1007/s11306-023-01975-2 36750484 PMC11238255

[23] He W, Marchuk H, Koeberl D, Kasumov T, Chen X, Zhang GF (2024) Fasting alleviates metabolic alterations in mice with propionyl-CoA carboxylase deficiency due to Pcca mutation. Commun Biol 7:659 doi:10.1038/s42003-024-06362-8 38811689 PMC11137003

[24] He W, Wang Y, Xie EJ, Barry MA, Zhang GF (2021) Metabolic perturbations mediated by propionyl-CoA accumulation in organs of mouse model of propionic acidemia. Mol Genet Metab 134:257–266 doi:10.1016/j.ymgme.2021.09.009 34635437

[25] Hofherr SE, Senac JS, Chen CY, Palmer DJ, Ng P, Barry MA (2009) Short-term rescue of neonatal lethality in a mouse model of propionic acidemia by gene therapy. Hum Gene Ther 20:169–180 doi:10.1089/hum.2008.158 19025475 PMC2922073

[26] Hommes FA, Kuipers JR, Elema JD, Jansen JF, Jonxis JH (1968) Propionicacidemia, a new inborn error of metabolism. Pediatr Res 2:519–524 doi:10.1203/00006450-196811000-00010 5727920

[27] Kasumov T, Cendrowski AV, David F, Jobbins KA, Anderson VE, Brunengraber H (2007) Mass isotopomer study of anaplerosis from propionate in the perfused rat heart. Arch Biochem Biophys 463:110–117 doi:10.1016/j.abb.2007.02.022 17418801 PMC2047339

[28] Kinman RP, Kasumov T, Jobbins KA, Thomas KR, Adams JE, Brunengraber LN, Kutz G, Brewer WU, Roe CR, Brunengraber H (2006) Parenteral and enteral metabolism of anaplerotic triheptanoin in normal rats. Am J Physiol Endocrinol Metab 291:E860–866 doi:10.1152/ajpendo.00366.2005 16705058

[29] Knottnerus SJG, Bleeker JC, Ferdinandusse S, Houtkooper RH, Langeveld M, Nederveen AJ, Strijkers GJ, Visser G, Wanders RJA, Wijburg FA, Boekholdt SM, Bakermans AJ (2020) Subclinical effects of long-chain fatty acid beta-oxidation deficiency on the adult heart: A case-control magnetic resonance study. J Inherit Metab Dis 43:969–980 doi:10.1002/jimd.12266 32463482 PMC7539973

[30] Kolker S, Garcia-Cazorla A, Valayannopoulos V, Lund AM, Burlina AB, Sykut-Cegielska J, Wijburg FA, Teles EL, Zeman J, Dionisi-Vici C, Baric I, Karall D, Augoustides-Savvopoulou P, Aksglaede L, Arnoux JB, Avram P, Baumgartner MR, Blasco-Alonso J, Chabrol B, Chakrapani A, Chapman K, EC IS, Couce ML, de Meirleir L, Dobbelaere D, Dvorakova V, Furlan F, Gleich F, Gradowska W, Grunewald S, Jalan A, Haberle J, Haege G, Lachmann R, Laemmle A, Langereis E, de Lonlay P, Martinelli D, Matsumoto S, Muhlhausen C, de Baulny HO, Ortez C, Pena-Quintana L, Ramadza DP, Rodrigues E, Scholl-Burgi S, Sokal E, Staufner C, Summar ML, Thompson N, Vara R, Pinera IV, Walter JH, Williams M, Burgard P (2015) The phenotypic spectrum of organic acidurias and urea cycle disorders. Part 1: the initial presentation. J Inherit Metab Dis 38:1041–1057 doi:10.1007/s10545-015-9839-3 25875215

[31] Kolker S, Valayannopoulos V, Burlina AB, Sykut-Cegielska J, Wijburg FA, Teles EL, Zeman J, Dionisi-Vici C, Baric I, Karall D, Arnoux JB, Avram P, Baumgartner MR, Blasco-Alonso J, Boy SP, Rasmussen MB, Burgard P, Chabrol B, Chakrapani A, Chapman K, Cortes ISE, Couce ML, de Meirleir L, Dobbelaere D, Furlan F, Gleich F, Gonzalez MJ, Gradowska W, Grunewald S, Honzik T, Horster F, Ioannou H, Jalan A, Haberle J, Haege G, Langereis E, de Lonlay P, Martinelli D, Matsumoto S, Muhlhausen C, Murphy E, de Baulny HO, Ortez C, Pedron CC, Pintos-Morell G, Pena-Quintana L, Ramadza DP, Rodrigues E, Scholl-Burgi S, Sokal E, Summar ML, Thompson N, Vara R, Pinera IV, Walter JH, Williams M, Lund AM, Garcia-Cazorla A (2015) The phenotypic spectrum of organic acidurias and urea cycle disorders. Part 2: the evolving clinical phenotype. J Inherit Metab Dis 38:1059–1074 doi:10.1007/s10545-015-9840-x 25875216

[32] Kor D, Seker-Yilmaz B, Bulut FD, Kilavuz S, Oktem M, Ceylaner S, Yildizdas D, Onenli-Mungan N (2019) Clinical features of 27 Turkish Propionic acidemia patients with 12 novel mutations. Turk J Pediatr 61:330–336 doi:10.24953/turkjped.2019.03.003 31916709

[33] Kott-Blumenkranz R, Pappas CT, Bensch KG (1981) A study of the ultrastructure of the organs and of cultured fibroblasts incubated with isoleucine from a patient with propionic acidemia. Hum Pathol 12:1141–1148 doi:10.1016/s0046-8177(81)80336-x 7333577

[34] Kovacevic A, Garbade SF, Horster F, Hoffmann GF, Goren o M, Mereles D, Kolker S, Staufner C (2022) Detection of early cardiac disease manifestation in propionic acidemia - Results of a monocentric cross-sectional study. Mol Genet Metab 137:349–358 doi:10.1016/j.ymgme.2022.10.007 36395710

[35] Liu X, Cooper DE, Cluntun AA, Warmoes MO, Zhao S, Reid MA, Liu J, Lund PJ, Lopes M, Garcia BA, Wellen KE, Kirsch DG, Locasale JW (2018) Acetate Production from Glucose and Coupling to Mitochondrial Metabolism in Mammals. Cell 175:502–513 e513 doi:10.1016/j.cell.2018.08.040 30245009 PMC6173642

[36] Maines E, Moretti M, Vitturi N, Gugelmo G, Fasan I, Lenzini L, Piccoli G, Gragnaniello V, Maiorana A, Soffiati M, Burlina A, Franceschi R (2023) Understanding the Pathogenesis of Cardiac Complications in Patients with Propionic Acidemia and Exploring Therapeutic Alternatives for Those Who Are Not Eligible or Are Waiting for Liver Transplantation. Metabolites 13 doi:10.3390/metabo13040563 PMC10143878 37110221

[37] Marchuk H, Wang Y, Ladd ZA, Chen X, Zhang GF (2023) Pathophysiological mechanisms of complications associated with propionic acidemia. Pharmacol Ther 249:108501 doi:10.1016/j.pharmthera.2023.108501 37482098 PMC10529999

[38] Mardach R, Verity MA, Cederbaum SD (2005) Clinical, pathological, and biochemical studies in a patient with propionic acidemia and fatal cardiomyopathy. Mol Genet Metab 85:286–290 doi:10.1016/j.ymgme.2005.04.004 15939644

[39] Martini WZ, Stanley WC, Huang H, Rosiers CD, Hoppel CL, Brunengraber H (2003) Quantitative assessment of anaplerosis from propionate in pig heart in vivo. Am J Physiol Endocrinol Metab 284:E351–356 doi:10.1152/ajpendo.00354.2002 12388135

[40] Miyazaki T, Ohura T, Kobayashi M, Shigematsu Y, Yamaguchi S, Suzuki Y, Hata I, Aoki Y, Yang X, Minjares C, Haruta I, Uto H, Ito Y, Muller U (2001) Fatal propionic acidemia in mice lacking propionyl-CoA carboxylase and its rescue by postnatal, liver-specific supplementation via a transgene. J Biol Chem 276:35995–35999 doi:10.1074/jbc.M105467200 11461925

[41] Reszko AE, Kasumov T, Pierce BA, David F, Hoppel CL, Stanley WC, Des Rosiers C, Brunengraber H (2003) Assessing the reversibility of the anaplerotic reactions of the propionyl-CoA pathway in heart and liver. J Biol Chem 278:34959–34965 doi:10.1074/jbc.M302013200 12824185

[42] Ribas GS, Manfredini V, de Marco MG, Vieira RB, Wayhs CY, Vanzin CS, Biancini GB, Wajner M, Vargas CR (2010) Prevention by L-carnitine of DNA damage induced by propionic and L-methylmalonic acids in human peripheral leukocytes in vitro. Mutat Res 702:123–128 doi:10.1016/j.mrgentox.2010.07.008 20659584

[43] Ribas GS, Manfredini V, de Mari JF, Wayhs CY, Vanzin CS, Biancini GB, Sitta A, Deon M, Wajner M, Vargas CR (2010) Reduction of lipid and protein damage in patients with disorders of propionate metabolism under treatment: a possible protective role of L-carnitine supplementation. Int J Dev Neurosci 28:127–132 doi:10.1016/j.ijdevneu.2010.01.002 20100562

[44] Rivera-Barahona A, Fulgencio-Covian A, Perez-Cerda C, Ramos R, Barry MA, Ugarte M, Perez B, Richard E, Desviat LR (2017) Dysregulated miRNAs and their pathogenic implications for the neurometabolic disease propionic acidemia. Sci Rep 7:5727 doi:10.1038/s41598-017-06420-8 28720782 PMC5516006

[45] Roginski AC, Cecatto C, Wajner SM, Camera FD, Castilho RF, Wajner M, Amaral AU (2019) Experimental evidence that maleic acid markedly compromises glutamate oxidation through inhibition of glutamate dehydrogenase and alpha-ketoglutarate dehydrogenase activities in kidney of developing rats. Mol Cell Biochem 458:99–112 doi:10.1007/s11010-019-03534-7 31032535

[46] Roginski AC, Zemniacak AB, Marschner RA, Wajner SM, Ribeiro RT, Wajner M, Amaral AU (2022) Disruption of mitochondrial functions involving mitochondrial permeability transition pore opening caused by maleic acid in rat kidney. J Bioenerg Biomembr 54:203–213 doi:10.1007/s10863-022-09945-4 35902433

[47] Salmi H, Leonard JV, Lapatto R (2012) Patients with organic acidaemias have an altered thiol status. Acta Paediatr 101:e505–508 doi:10.1111/j.1651-2227.2012.02799.x 22849335

[48] Storgaard JH, Madsen KL, Lokken N, Vissing J, van Hall G, Lund AM, Orngreen MC (2020) Impaired lipolysis in propionic acidemia: A new metabolic myopathy? JIMD Rep 53:16–21 doi:10.1002/jmd2.12113 32395405 PMC7203654

[49] Tajima G, Kagawa R, Sakura F, Nakamura-Utsunomiya A, Hara K, Yuasa M, Hasegawa Y, Sasai H, Okada S (2021) Current Perspectives on Neonatal Screening for Propionic Acidemia in Japan: An Unexpectedly High Incidence of Patients with Mild Disease Caused by a Common PCCB Variant. Int J Neonatal Screen 7 doi:10.3390/ijns7030035 PMC8293189 34203287

[50] Tomcik K, Ibarra RA, Sadhukhan S, Han Y, Tochtrop GP, Zhang GF (2011) Isotopomer enrichment assay for very short chain fatty acids and its metabolic applications. Anal Biochem 410:110–117 doi:10.1016/j.ab.2010.11.030 21112315 PMC3055175

[51] Wang Y, Christopher BA, Wilson KA, Muoio D, McGarrah RW, Brunengraber H, Zhang GF (2018) Propionate-induced changes in cardiac metabolism, notably CoA trapping, are not altered by l-carnitine. Am J Physiol Endocrinol Metab 315:E622–E633 doi:10.1152/ajpendo.00081.2018 30016154 PMC6230704

[52] Wang Y, Yang H, Geerts C, Furtos A, Waters P, Cyr D, Wang S, Mitchell GA (2023) The multiple facets of acetyl-CoA metabolism: Energetics, biosynthesis, regulation, acylation and inborn errors. Mol Genet Metab 138:106966 doi:10.1016/j.ymgme.2022.106966 36528988

[53] Wang Y, Zhu S, He W, Marchuk H, Richard E, Desviat LR, Young SP, Koeberl D, Kasumov T, Chen X, Zhang GF (2024) The attenuated hepatic clearance of propionate increases cardiac oxidative stress in propionic acidemia. Basic Res Cardiol doi:10.1007/s00395-024-01066-w PMC11702364 38992300

[54] Wilson KA, Han Y, Zhang M, Hess JP, Chapman KA, Cline GW, Tochtrop GP, Brunengraber H, Zhang GF (2017) Inter-relations between 3-hydroxypropionate and propionate metabolism in rat liver: relevance to disorders of propionyl-CoA metabolism. Am J Physiol Endocrinol Metab 313:E413–E428 doi:10.1152/ajpendo.00105.2017 28634175 PMC5668600

[55] Wolf B, Hsia YE, Sweetman L, Gravel R, Harris DJ, Nyhan WL (1981) Propionic acidemia: a clinical update. J Pediatr 99:835–846 doi:10.1016/s0022-3476(81)80004-2 7031206

[56] Wongkittichote P, Ah Mew N, Chapman KA (2017) Propionyl-CoA carboxylase - A review. Mol Genet Metab 122:145–152 doi:10.1016/j.ymgme.2017.10.002 29033250 PMC5725275

[57] Zhang GF, Jensen MV, Gray SM, El K, Wang Y, Lu D, Becker TC, Campbell JE, Newgard CB (2021) Reductive TCA cycle metabolism fuels glutamine- and glucose-stimulated insulin secretion. Cell Metab 33:804–817 e805 doi:10.1016/j.cmet.2020.11.020 33321098 PMC8115731

[58] Zhang Y, Agarwal KC, Beylot M, Soloviev MV, David F, Reider MW, Anderson VE, Tserng KY, Brunengraber H (1994) Nonhomogeneous labeling of liver extra-mitochondrial acetyl-CoA. Implications for the probing of lipogenic acetyl-CoA via drug acetylation and for the production of acetate by the liver. J Biol Chem 269:11025–11029 8157628

[59] Zhang Y, Peng C, Wang L, Chen S, Wang J, Tian Z, Wang C, Chen X, Zhu S, Zhang GF, Wang Y (2023) Prevalence of propionic acidemia in China. Orphanet J Rare Dis 18:281 doi:10.1186/s13023-023-02898-w 37689673 PMC10493020

